# Libraries for two-hybrid screening of yeast and hyphal growth forms in *Zymoseptoria tritici*^☆^

**DOI:** 10.1016/j.fgb.2015.03.023

**Published:** 2015-06

**Authors:** W. Ma, S. Kilaru, C. Collins, M. Courbot, G. Steinberg

**Affiliations:** a Biosciences, University of Exeter, Exeter EX4 4QD, UK; b Syngenta Crop Protection AG, Schaffhauserstrasse, 4332 Stein, Switzerland; c Shannon Applied Biotechnology Centre, Limerick Institute of Technology, Moylish Park, Limerick, Ireland^2^

**Keywords:** Protein–protein interaction, Pathogenic fungi, Wheat disease, Septoria tritici blotch, *Mycosphaerella graminicola*, Y2H, yeast two hybrid, DNA, deoxyribonucleic acid, cDNA, complementary deoxyribonucleic acid, gDNA, genomic deoxyribonucleic acid, RNA, ribonucleic acid, *mcs1*, myosin-chitin synthase 1, *rab7* and *rab11*, small GTPases, *cpy1*, vacuolar carboxypeptidase Y, *mdm10*, mitochondrial distribution and morphology protein 10, *atg8* and *atg4*, autophagy-related proteins, OD, optical density, bp, base pairs, cfu, colony-forming unit

## Abstract

Pathogenic fungi are constantly emerging resistance to anti-fungal treatments. Therefore, identification of new fungicide targets is important. Good candidates are essential fungal proteins and their regulators. An efficient way to reveal the molecular environment of an essential protein is the search for interacting factors. Here, we establish three yeast two-hybrid libraries, covering yeast and hyphal stages of the wheat pathogen *Zymoseptoria tritici*. No detectable genomic DNA was present in any of the 3 libraries. Random amplification revealed that the libraries include cDNA fragments of up to 2000 bp, suggesting that small-to-medium sized proteins are represented therein. Indeed, full-length cDNAs of five proteins were found in all libraries. The full-length cDNA of large chitin synthase gene *mcs1* (5742 bp with introns; 5568 bp without introns) could not be amplified, but its 5′ and 3′ regions were represented, suggesting that even larger genes are covered in all libraries. Finally, we tested for the expected interaction of the autophagy proteins ZtAtg4 and ZtAtg8 in *Z. tritici*, and then used ZtAtg4 to screen one of the two-hybrid libraries. Indeed, we found ZtAtg8 as a positive interaction partner, confirming that interacting proteins can be identified. Thus, these molecular tools promise to be useful in identifying novel fungicide target proteins.

## Highlights

• We provide three yeast two hybrid libraries for use in *Z. tritici.*
• Quality control experiments confirm the presence of cDNA insert of up to 2000 bp.
• Using Atg4 and Atg8 we show that the libraries are useful to identify protein interactions.

## 1 Introduction

Yeast-two hybrid (Y2H) methods provide a powerful approach to obtain binary protein–protein interaction information. In recent years, high-throughput Y2H maps were generated for a large range of model organisms, including the yeast *Saccharomyces cerevisiae* (Fromont-Racine et al., 1997; Ito et al., 2001; Uetz et al., 2000), fruit flies and worms (Giot et al., 2003; Reboul et al., 2003; Walhout et al., 2000), plants (Arabidopsis Interactome Mapping, 2011) and humans (Colland et al., 2004; Rual et al., 2005; Stelzl et al., 2005); overview in (Bruckner et al., 2009). In filamentous fungi, Y2H techniques have been widely used to widen our understanding of specific cellular processes, including endocytosis in *Aspergillus oryzae* (Matsuo et al., 2013), motor protein function (Seidel et al., 2012), pH signalling in *Aspergillus nidulans* (Herranz et al., 2005; Galindo et al., 2007) and biogenesis of peroxisome derived glyoxysomes and Woronin bodies in *Neurospora crassa* (Managadze et al., 2010). Y2H techniques have also provided insight into fungal plant pathology, such as in the identification of factors involved in appressorium formation in *Magnaporthe grisea* (Kulkarni and Dean, 2004) and *Ustilago maydis* (Lanver et al., 2010).

The Y2H system was developed by Fields and Song (1989). It makes use of the yeast GAL4 protein, which contains a DNA-binding domain and transcriptional activation domain (Keegan et al., 1986). Here, one “bait” protein is fused to “the Gal4” DNA-binding domain, whereas the other putative interaction partner, the “prey”, is fused to the GAL4 activation domain (Stynen et al., 2010). When both recombinant proteins are expressed in an appropriate *S. cerevisiae* strain, bait and prey proteins can physically interact. This act reconstitutes the functional transcription factor and leads to the expression of reporter genes. These reporters can be auxotrophy markers, antibiotic resistance conveyors or colour indicators, which are usually combined to increase the accuracy of the system (Stephens and Banting, 2000). Principally, the Y2H system can be used in several ways. In a targeted approach, the putative interaction of two proteins, used as bait and prey, is directly investigated. In order to identify novel interacting partners, a library screening approach is used to identify pair wise interactions between a defined protein of interest (bait) and interacting partners (potential prey) present in cDNA libraries (Bruckner et al., 2009). This powerful approach has recently led to the identification of fungal and plant proteins during host-pathogen interaction in *U. maydis* (Mueller et al., 2013) and *Fusarium graminearum* (Niu et al., 2013).

Here, we introduce two vectors and three cDNA libraries, suitable to undertake targeted Y2H interaction studies and Y2H screening for interacting proteins in the wheat pathogen *Zymoseptoria tritici*. The libraries cover yeast-like and hyphal cells of the fully sequenced wild-type strain of IPO323 (Goodwin et al., 2011; Kema and van Silfhout, 1997) and a combination of both in a second wild-type strain K4418 (provided by Syngenta, Jealott’s Hill, UK). We show that cDNA fragments of up to 2000 bp are represented in the libraries. We tested the interaction of ZtAtg4, and ZtAtg8, two proteins involved in autophagy that have been shown to interact in *Magnaporthe oryzae* (Liu et al., 2010). Finally, we used ZtAtg4 as bait and screened our library for interacting partners. Indeed, we found the expected ZtAtg8 in the Y2H library.

## 2 Materials and methods

### 2.1 Yeast strains

To construct the Y2H libraries, the yeast strain Y187 (*MAT*α, *ura3-52*, *his3-200*, *ade2-101*, *trp1-901*, *leu2-3*, *112*, *gal4Δ*, *gal80Δ*, *met–*, *URA3:: GAL1*UAS*–Gal1*TATA*–LacZ*, *MEL1)* was used (Harper et al., 1993), which contains the auxotrophic markers *Trp1* (required for tryptophan biosynthesis) and *Leu2* (required for leucine biosynthesis), as well as the reporter genes *MEL1* (produces the enzyme α-galactosidase which can be detected by addition chromogenic substrate X-α-gal) and *LacZ* (produces the enzyme β-galactosidase which can be detected by addition chromogenic substrate X-α-gal). Bait vectors were transformed into yeast strain Y2HGold (*MATa*, *trp1-901*, *leu2-3*, *112*, *ura3-52*, *his3-200*, *gal4Δ*, *gal80Δ*, *LYS2:: GAL1_UAS_–Gal1_TATA_–His3*, *GAL2_UAS_–Gal2_TATA_–Ade2*, *URA3:: MEL1_UAS_–Mel1_TATA_*, *AUR1-C MEL1*) was used. This strain contains the auxotrophic markers *Trp1* and *Leu2*, as well as the reporter genes *MEL1*, *LacZ*, *AbA^r^* (confers strong resistance to toxic drug Aureobosidin A), *HIS3* (required for histidine biosynthesis) and *ADE2* (required for adenine biosynthesis).

### 2.2 Fungal strains and growth conditions

The fully sequenced *Z. tritici* wild-type isolate IPO323 (Goodwin et al., 2011; Kema and van Silfhout, 1997) and another wild-type isolate K4418 (provided by Syngenta, Jealott’s Hill, UK) were used in this study. The isolate was inoculated from stocks stored in glycerol at −80 °C onto solid YPD agar (yeast extract, 10 g/l; peptone, 20 g/l; glucose, 20 g/l; agar, 20 g/l) and grown at 18 °C for 4–5 days. In order to induce the yeast-like growth of IPO323, the cells were cultivated in YG (yeast extract, 10 g/l; glucose 30 g/l) media at 18 °C with 200 rpm for two days, while induction of the hyphal growth of IPO323 was achieved by cultivating the cells in YG media at 25 °C with 100 rpm for two days. In order to induce both yeast-like and hyphal growth of K4418, the cells were cultivated in YEPS (1% yeast extract, 2% bacto-peptone, 2% sucrose; (Tsukuda et al., 1988) media at 18 °C with 200 rpm for three days.

### 2.3 Microscopy

Yeast-like cells, hyphal cells and mixed yeast-like and hyphal cells were induced as described above and placed onto a 2% agar cushion for direct observation. DIC imaged were acquired using a motorized inverted microscope (IX81; Olympus, Hamburg, Germany), equipped with an UPlanSApo 60X/1.35 Oil objective (Olympus, Hamburg, Germany).

### 2.4 RNA isolation

The total RNA was isolated from yeast-like cells of IPO323, hyphal cells of IPO323 and both yeast-like and hyphal cells of K4418. To this end, the *Z. tritici* isolates were grown as described above and cells were harvested by centrifugation at 3000 rpm for 10 min. Sedimented cells were ground in liquid nitrogen with a mortar and pestle and total RNA was extracted according to the manufactures instructions of RNeasy plant mini kit (Qiagen, Manchester, UK). The RNA samples were treated with DNase I (Life Technologies, Paisley, UK) and the removal of genomic DNA contamination was confirmed by PCR amplification of 5′ end of the *mcs1* gene with CC-125 and CC-117 primers (Table 1).

### 2.5 First-strand cDNA synthesis for library construction

Approximately 2 μg of DNase I treated RNA was reverse transcribed into cDNA using the oligo dT primer CDS III (Table 1) and the enzyme SMART MMLV Reverse Transcriptase according to Make Your Own “Mate & Plate™” Library System User Manual (Clontech, Saint-Germain-en-Laye, France). SMART III-modified oligo (Table 1) was attached as an anchor at the 5′ end of transcripts. The successful first-strand cDNA synthesis was confirmed by PCR with CC-125 and CC-117 primers (Table 1) and DreamTaq DNA Polymerase (Thermo Scientific, Leicestershire, UK).

### 2.6 Double stranded cDNA synthesis

The double strand cDNA was synthesized using the obtained single strand cDNA above and Advantage 2 Polymerase Mix (Clontech, Saint-Germain-en-Laye, France), all in a total volume of 100 μl. Initially, 2 μl First-Strand cDNA, 70 μl Deionized H_2_O, 10 μl 10× Advantage 2 PCR Buffer, 2 μl 50× dNTP Mix, 2 μl 5′ PCR Primer, 2 μl 3′ PCR Primer, 10 μl 10× Melting Solution, 2 μl 50× Advantage 2 Polymerase Mix were added to 0.2 ml PCR tubes and thermal reaction was carried out. The cycling parameters were 95 °C for 30 s followed by 24 cycles of 95 °C for 10 s, 68 °C for 6 min (programmed to increase the extension time by 5 s with each successive cycle) and 68 °C for 5 min.

### 2.7 Purification of cDNA

In order to remove the fragmented cDNA, smaller than 200 bp, the double stranded cDNA samples were purified by using CHROMA SPIN TE-400 columns (Clontech, Saint-Germain-en-Laye, France). The equilibration buffer in the Chroma spin columns were removed by centrifuged at 700*g* for 5 min. Subsequently, the entire cDNA preparation was loaded to the centre of the column and centrifuged at 700*g* for 5 min. The flow-though was carefully collected into the collection tube and the cDNA was precipitated with 1/10th volume of 3 M sodium acetate, 2.5 volumes of ice-cold 100% ethanol and incubated at −20 °C for 60 min. The tubes were centrifuged at 14,000 rpm for 20 min and supernatant was discarded. The cDNA pellet was air dried and resuspended in 20 μl water.

### 2.8 Two-hybrid library construction using cDNA

The above column purified double-stranded cDNA was used for two-hybrid library construction using *in vivo* recombination in yeast strain Y187 and the Matchmaker® Gold Yeast Two-Hybrid System (Clontech, Saint-Germain-en-Laye, France). In order to prepare the *S. cerevisiae* competent cells, one colony of strain Y187 (freshly grown) was grown in 3 ml YPDA (yeast extract, 10 g/l; peptone, 20 g/l; glucose, 20 g/l; 15 ml of a 0.2% adenine hemisulfate/l) medium at 30 °C at 250 rpm for 8–12 h. Five μl of the culture was transferred to 50 ml YPDA medium and grown until the OD_600_ reached 0.15–0.3 (16–20 h). The cells were harvested by centrifugation at 700*g* for 5 min and the pellet was resuspended in 100 ml YPDA medium and grown until the OD_600_ reaches to 0.4–0.5 (3–5 h). After another round of centrifugation, cells were washed once with 60 ml water and followed by a wash with 3 ml of 1.1×TE/LiAc and finally the cell pellet was resuspended in 1.2 ml of 1.1×TE/LiAc, which was used for yeast transformation.

The column-purified double stranded cDNA was cloned into pGADT7^rec^ by using *in vivo* recombination in yeast. To this end, 20 μl double stranded cDNA (2–5 μg), 6 μl pGADT7^rec^ vector (digested with *Sma* I) (3 μg), 20 μl yeast maker carrier DNA (10 μg/μl), 600 μl of the above competent cells, 2.5 ml PEG/LiAc were added to 15 ml tube and incubated at 30 °C for 45 min. Then 160 μl DMSO was added and incubated at 42 °C for 20 min. The cells were harvested by centrifugation at 700*g* for 5 min and were resuspended in 3 ml YPD Plus medium (Clontech, Saint-Germain-en-Laye, France), followed by incubation at 30 °C for 90 min with gentle shaking. After another round of centrifugation, the sedimented cells were resuspended in 15 ml 0.9% (w/v) NaCl solution. Aliquots of 150 μl were spread on synthetically defined medium without Leucine (SD/-Leu) agar plates (145 cm diameter; yeast nitrogen base without amino acids, 6.9 g/l; glucose, 20 g/l; agar, 20 g/l; CSM/-Leu, 0.69 g/l (Formdeium, Norfolk, UK), pH-5.8) and incubated at 30 °C for 3–4 days. In parallel, 1/10 and 1/100 dilutions were also spread on SD/-Leu plates to determine the transformation efficiency. In order to harvest the pool of transformants, the plates were chilled at 4 °C for 3–4 h. Then five ml of freezing medium (a sterile suspension of 100 ml YPDA (Clontech, Saint-Germain-en-Laye, France) and 50 ml 75% glycerol) and few 4 mm sterile glass balls (Fisher Scientific, Loughborough, UK) were added to each plate to detach the colonies from the plate. Likewise, the cells were pooled from all 100 plates and stored at −80 °C as 1 ml aliquots.

### 2.9 PCR amplification of randomly chosen colonies

In order to check that cDNA molecules were successfully ligated with pGADT7^rec^ Vector, colony PCR was performed with 16 different colonies of each two-hybrid library using matchmaker Insert check PCR Mix 2 (Clontech, Saint-Germain-en-Laye, France). The PCR mix contains the primers, which bind either side of the multiple cloning site and thus allows detection of cDNA fragment sizes. The cycling parameters were 94 °C for 1 min followed by 30 cycles of 98 °C for 10 s and 68 °C for 3 min.

### 2.10 Identification of *Z. tritici* proteins and bioinformatics

To identify *Z. tritici* homologues of *rab7*, *rab11*, *cpy1*, and *mdm10*, we downloaded he sequences of the respective sequences from *U. maydis* (accession numbers: Rab 7, XP_761658.1; Rab 11, XP_757798.1; (Fuchs and Steinberg, 2005), *Candida albicans* (Cpy1: EAK92457.1, (Mukhtar et al., 1992)) and *S. cerevisiae* (Mdm10: A7A0F9.1; (Sogo and Yaffe, 1994)) from the NCBI server (http://www.ncbi.nlm.nih.gov/pubmed) and screened the published genome of *Z. tritici* using BLASTP (http://genome.jgi.doe.gov/Mycgr3/Mycgr3.home.html). ZtAtg4 and ZtAtg8 were identified in the same way, using the predicted sequences for Atg4 and Atg8 from *M. oryzae* (accession numbers: ACJ06587.1 and ACJ06588.1, respectively). The length of the predicted *Z. tritici* proteins was confirmed by comparing all sequences against the published fungal counterparts using ClustalW (http://www.ebi.ac.uk/Tools/msa/clustalw2/). This indicated that ZtAtg8 was wrongly annotated and we used a shorter version, deleted in the N-terminal 432 amino acids. All sequences were compared against their fungal homologues using EMBOSS Needle (http://www.ebi.ac.uk/Tools/psa/emboss_needle/). The domain structures were analyzed in PFAM (http://pfam.xfam.org/search/sequence).

### 2.11 PCR amplification of the cDNA of interest from the two-hybrid libraries

In order to amplify the cDNA of interest from the two-hybrid libraries, the 1-ml aliquots of each libraries were grown in 15 ml SD/-Leu media at 30 °C with 250 rpm for 12 h and the plasmid DNA was isolated using phenol:chloramphenicol:isoamylalcohol as described in Kilaru and Steinberg (2015) and 1 μl DNA was used for PCR. The full length cDNAs of *rab7* (amplified with SK-Sep-63 and SK-Sep-64; Table 1), *rab11* (amplified with SK-Sep-65 and SK-Sep-66; Table 1), *atg8* (amplified with SK-Sep-67 and SK-Sep-68; Table 1), *cpy1* (amplified with SK-Sep-73 and SK-Sep-74; Table 1), *mdm10* (amplified with SK-Sep-75 and SK-Sep-76; Table 1); 5′ end of *mcs1* cDNA fragment (amplified with CC-125 and CC-117; Table 1), 3′ end of *mcs1* cDNA fragment (amplified with CC-113 and CC-161; Table 1) using high fidelity Phusion DNA polymerase (Thermo Scientific, Leicestershire, UK). The cycling parameters were 94 °C for 2 min followed by 35 cycles of 94 °C for 20 s, 60 °C for 20 s, 72 °C for 1 min/kb and final extension at 72 °C for 10 min. In order to get the sufficient PCR product, a second round of PCR was performed by using the initial PCR product as DNA template with the identical cycling conditions. The DNA bands of interest were excised from the gel and purified by using silica glass suspension as described previously (for further details see Kilaru and Steinberg, 2015). The purified DNA fragments were cloned into StrataClone PCR Cloning Vector pSC-A-amp/kan (Agilient Technologies, Santa Clara, CA, USA) by using T4 DNA ligase (New England Biolabs, Herts, UK). The ligation mixture was transformed into Strataclone solopack competent cells (Agilient Technologies, Santa Clara CA, USA) and the recombinant *Escherichia coli* colonies were selected on DYT agar media supplemented with 100 μg/ml Ampicillin. The corresponding plasmids were isolated from the *E. coli* cells as described in Kilaru and Steinberg (2015). The plasmid DNA was further purified by using Wizard® SV Gel and PCR Clean-Up System (Promega, Southampton, UK) and DNA sequencing was carried out at GATC biotech (GATC biotech, Cologne, Germany).

### 2.12 Construction of “bait” and “prey” vectors

The full-length *Z. tritici* cDNAs of *ZtAtg4* (1317 bp) and *ZtAtg8* (360 bp) were amplified from the plasmid DNA isolated from the Y2H library (IPO323_Yeasts) using high fidelity Phusion DNA polymerase (Thermo Scientific, Leicestershire, UK). Primers SK-Sep-258 and SK-Sep-259 (Table 1) were used to amplify cDNA of *ZtAtg4*, while primers WM-3 and WM-4 (Table 1) were used to amplify cDNA of *ZtAtg8* (Table 1). The amplified PCR products were loaded on to 1% agarose gel and purified as described before (for further details see Kilaru and Steinberg, 2015). The purified *ZtAtg4* and *ZtAtg8* cDNAs were cloned into vectors *Bam*HI and *Eco*RI digested pGBKT7 and *Bam*HI and *Eco*RI digested pGADT7^rec^ respectively (Clontech, Saint-Germain-en-Laye, France) by using In-Fusion HD cloning kit (Clontech, Saint-Germain-en-Laye, France) resulting in pGBKT7ZtAtg4 (bait vector) and pGADT7^rec^_ZtAtg8 (prey vector) respectively. The presence of ZtAtg4 and ZtAtg8 in the vectors pGBKT7ZtAtg4 and pGADT7^rec^_ZtAtg8 were confirmed by DNA sequencing at GATC Biotech (GATC Biotech, Cologne, Germany). In order to detect the interaction of ZtAtg4 and ZtAtg8, both pGBKT7ZtAtg4 (bait vector) and pGADT7^rec^_ZtAtg8 (prey vector) were co-transformed into yeast strain Y2HGold (Clontech, Saint-Germain-en-Laye, France) and transformants were selected on SD/-Trp/-Leu agar plates (yeast nitrogen base without amino acids, 6.9 g/l; glucose, 20 g/l; agar, 20 g/l; CSM/-Leu/-Trp, 0.64 g/l (Formedium, Norfolk, UK), pH-5.8). For control experiments, plasmids GBKT7ZtAtg4 and pGADT7^rec^; pGBKT7 and pGADT7^rec^_ZtAtg8; pGBKT7 and pGADT7^rec^ were co-transformed into yeast strain Y2HGold with the selection of transformants on SD/-Trp/-Leu agar plates.

### 2.13 Two-hybrid library screening

To screen Y2H library, using ZtAtg4 as a “bait” protein, the yeast strain Y2HGold containing plasmid pGBKT7_ZtAtg4 was grown in 50 ml SD/-Trp at 30 °C with 250 rpm until the OD_600_ reaches 0.8 (16–20 h). The cells were harvested by centrifugation 1000*g* for 5 min and cell density was adjusted to >1 × 10^8^ cells per ml in SD/-Trp. Five ml of the bait strain cell culture and one ml of the library strain Y187 cell culture (one aliquot of the two-hybrid library) were added to 45 ml of 2× YPDA containing 50 μg/ml kanamycin and cultivated at 30 °C for at 50 rpm, until zygotes appear, which was checked microscopically after 20–24 h. Cells were harvested by centrifugation at 1000*g* for 5 min, the cell pellet was washed with 0.5× YPDA containing 50 μg/ml kanamycin and finally resuspended in 10 ml containing 50 μg/ml kanamycin. In order to calculate the mating efficiency, 100 μl of this mated culture (1/10, 1/100, 1/1000 and 1/10,000 dilutions) was spread on to SD/-Trp, SD/-Leu, and SD/-Trp/-Leu agar plates and incubated at 30 °C for 3–5 days. The remaining culture was plated on to SD/-Trp/-Leu/X-α-Gal/AbA (40 μg/ml X-α-Gal and 200 ng/ml Aureobasidin A) agar plates. In total 50 plates, 145 cm diameter, were used (200 μl per plate) and incubated at 30 °C for 3–5 days. The blue colonies appeared on SD/-Trp/-Leu/X-α-Gal/AbA agar plates were touched with 200 μl tip and streaked on to high stringency plates SD/-Ade/-His/-Trp/-Leu/X-α-Gal/AbA (yeast nitrogen base without amino acids, 6.9 g/l; glucose, 20 g/l; agar, 20 g/l; CSM/-Ade/-His/-Leu/-Trp, 0.61 g/l (Formdeium, Norfolk, UK), pH-5.8, 40 μg/ml X-α-Gal and 200 ng/ml Aureobasidin A) and incubated at 30 °C for 3–5 days. For the control experiments, yeast strain Y2HGold containing plasmids GBKT7ZtAtg4 and pGADT7^rec^; pGBKT7 and pGADT7^rec^_ZtAtg8; pGBKT7 and pGADT7^rec^ were grown in SD/-Trp/-Leu media for 24 h and plated on to SD/-Trp/-Leu agar media and SD/-Ade/-His/-Trp/-Leu/X-α-Gal/AbA agar media and incubated at 30 °C for 3–5 days.

## 3 Results and discussion

### 3.1 Preparing cDNA from yeast-like and hyphal cells of *Z. tritici*

**Fig. 1:**
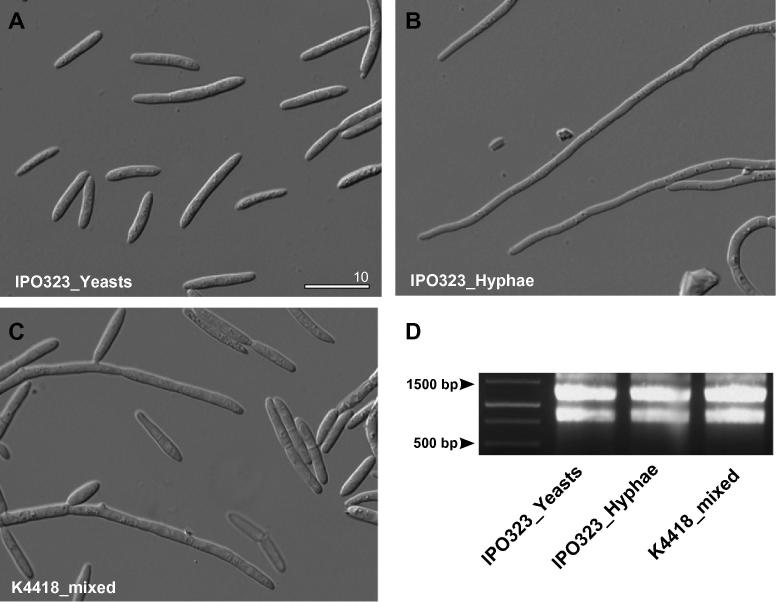
RNA purification from *Z. tritici* cells grown in liquid culture. Total RNA was purified from 2 to 3 day old cultures of strain IPO323 and strain K4418, grown under conditions that induce yeast-like growth (A; IPO323_Yeasts), hyphal growth (B; IPO323_Hyphae), or that induces both growth forms (C; K4418_mixed). (D) A gel shows purification of total RNA from cells harvested from all three culture conditions. Sizes are indicated.

**Fig. 2:**
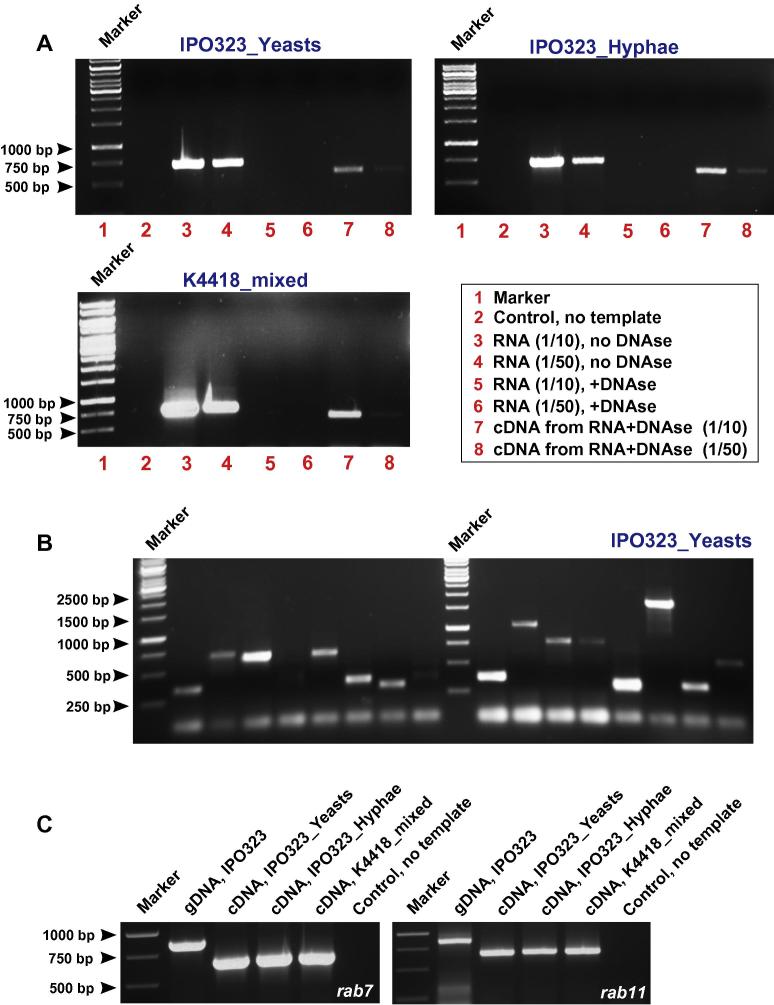
Agarose gels showing the outcome of control PCR experiments. (A) DNA fragments of 5′ end of the myosin chitin synthase 1 (*mcs1*) were amplified using primers CC-125 and CC-117 (see Table 1). In all three preparations, no PCR fragment was found in the absence of template (control), whereas strong bands of 707 bp appeared after PCR on total RNA preparations (lanes 3 and 4). These bands were not present when RNA which had been pre-treated with DNase I to remove contaminating genomic DNA (lanes 5 and 6). After transcribing this purified RNA into cDNA, PCR product of 585 bp appeared confirming the splicing of 122 bp predicted intron. The absence of 122 bp intron on the cDNA product was further confirmed by cDNA sequencing. Note that (1/10) and (1/50) indicate dilutions (1/10: 1 part RNA, 9 parts water; 1/50: 1 part RNA, 49 parts water). (B) Random amplification of yeast colonies with match maker PCR mix generated products with maximum sizes of 2000 bp in all three cDNA libraries (only IPO323_Yeasts shown). This suggests that entire open reading frames of proteins, up to ∼600–700 aa long, are represented in the library. Note that PCRs designed to amplify shorter fragments (585 bp and 1544 bp) of the chitin synthase gene *mcs1* (5568 bp without introns) still produced positive bands (see main text). This suggests that fragments of larger genes are also represented in the libraries. (C) Primers were designed to amplify the entire open reading frame of the small GTPases *rab7* (815 bp) and *rab11* (807 bp) (see Table 1, *rab7*: primers SK-Sep-63 and SK-Sep-64; *rab11*: primers SK-Sep-65 and SK-Sep-66). Both open reading frames were amplified from genomic DNA of IPO323. Smaller fragments (615 bp and 633 bp) were found after PCR reactions using cDNA from all three preparations (IPO323_Yeasts, IPO323_Hyphae, K4418_mixed). This corresponds with the predicted presence of introns in both genes (*rab7*: 815 bp; *rab11*: 807 bp; see main text for more details) and further confirmed by DNA sequencing.

*Z. tritici* is a dimorphic fungus that grows in a yeast-like form, but upon changing the temperature or nutritional status, switches to hyphal growth (Mehrabi et al., 2006; Motteram et al., 2011). In order to obtain mRNA from predominantly yeast-like cells and hyphal forms of IPO323, we grew the cells at 18 °C and 24 °C in a complete medium (see Section 2 for details). Microscopic investigation of the cells after two days confirmed that these growth conditions enriched yeast cells and hyphae, respectively (Fig. 1A and B). The isolate K4418 was grown for three days in conditions that led to growth of yeast-like and hyphal cells (Fig. 1C). We isolated total RNA from the above cultures and its integrity was confirmed by agarose gel electrophoresis (Fig. 1D). Genomic DNA contamination was removed from the RNA by DNase I treatment (see Section 2). The removal of genomic DNA contamination from all total RNA preparations was confirmed by polymerase chain reaction (PCR) using primers, specific for a 5′ end of 707 bp fragment of the *Z. tritici* chitin synthase gene *mcs1* (NCBI accession number: XP_003855029.1; see Table 1 for primer sequences CC-125 and CC-117). Electrophoresis showed that a DNA band of the expected size was amplified from total RNA (Fig. 2A, lane 3 and 4 in all three sub-panels; two dilutions are shown). After DNase I treatment, no band was amplified, demonstrating the absence of genomic DNA contaminations (Fig. 2A, lane 5 and 6 in all three sub-panels; two dilutions are shown). When these purified RNA samples were reverse transcribed into first strand cDNA (see Section 2) and PCR was performed, the DNA bands reappeared (Fig. 2A, lane 7 and 8 in all three sub-panels; two dilutions are shown), yet at a slightly smaller size, compared to the band amplified from genomic DNA. We speculate that this is due to the lack of a 122 bp intron, predicted in the *mcs1* gene (http://genome.jgi-psf.org/Mycgr3/Mycgr3.home.html). Indeed, DNA sequencing of the PCR products from all three libraries confirmed the absence of this intron in the amplicon. This indicated successful first-strand cDNA synthesis from the “yeast-like”, “hyphal” and “mixed morphology” *Z. tritici* total RNA.

### 3.2 Two-hybrid library construction

Next, we generated cDNA libraries using the Matchmaker® Gold Yeast Two-Hybrid System (Clontech, Saint-Germain-en-Laye, France; for details see Section 2). Positive interaction of bait and prey is monitored by four reporters, which include two auxotrophic reporters for histidine and adenine biosynthesis for growth selection, an α-galactosidase reporter which induces blue colonies and invokes resistance to the anti-fungal peptide Aureobasidin A. In a first step, we converted the single stranded cDNAs into double-stranded cDNAs, which were cloned into vector pGADT7^rec^ following manufacturer’s protocol (see Section 2) using *in vivo* recombination in yeast strain Y187 (Clontech, Saint-Germain-en-Laye, France). In this way, we constructed three different cDNA libraries, the 2–5 μg of double-stranded cDNA derived from the RNA isolated from yeast-like and hyphal cells of IPO323 and a mixed culture of K4418. In the following, we refer to these libraries as IPO323_Yeasts, IPO323_Hyphae and K4418_mixed, respectively. After transformation into the yeast strain Y187, we determined the transformation efficiencies within the range of 6.0 × 10^5^ to 7.0 × 10^5^ cfu/μg DNA. As each library was derived from 2 to 5 μg of cDNA, this result suggests that all the libraries contain more than one million independent colonies. This provided a first indication for a successful preparation of the Y2H libraries (manufacturer’s information, Clontech, Saint-Germain-en-Laye, France).

### 3.3 Validation of yeast two-hybrid libraries

The quality of the prepared Y2H libraries was further evaluated by using random PCR and gene specific PCR approaches. In a first test, 16 different colonies were randomly selected for each library and PCR was performed using Matchmaker insert PCR mix 2, which allows PCR amplification of plasmid-containing DNA directly from yeast colonies (Clontech, Saint-Germain-en-Laye, France). This analysis revealed that all plasmids contain inserts ranging in size between 300 bp and 2000 bp (Fig. 2B, only the outcome for testing IPO323_Yeasts is shown). We next aimed to amplify full-length cDNAs of interest. To this end, plasmid DNA was isolated from all three libraries and PCR was performed by using gene specific primers (Table 1). We choose genes for *Z. tritici* homologues of the small GTPases *rab7* (XP_003854495.1) and *rab11* (XP_003855659.1; 78% sequence identity with Rab7 and 75.1% sequence identity with Rab11 from *U. maydis*, respectively*)*, the vacuolar carboxypeptidase Y gene *cpy1* (XP_003854848.1; 56.9% identity to Cpy1 from *C. albicans*) and the gene encoding the mitochondrial distribution and morphology protein 10, *mdm10* (XP_003854898.1; 28.8% identity to Mdm10p from *S. cerevisiae*). All cDNAs were amplified using specific primers (see Table 1 and Section 2) from all three libraries (Fig. 2C, only *rab7* and *rab11* amplifications are shown). The PCR products were of the expected sizes (618 bp and 636 bp) and their identity was further confirmed by DNA sequencing. We found size differences for *rab7*, *rab11*, *atg8* and *cpy1*, when PCR was performed on genomic DNA and cDNA, suggesting that all genes contain introns (Fig. 2C, only *rab7* and *rab11* shown). Indeed, sequencing the PCR products from genomic and cDNA confirmed differences in sizes of the open reading frames (*rab7*: 618 bp cDNA, 815 bp gDNA; *rab11*: 636 bp cDNA, 807 bp gDNA; *atg8*: 360 bp cDNA, 512 bp gDNA; *cpy1*: 1,650 bp cDNA, 1,704 bp gDNA), whereas *mdm10* showed no difference in length (1179 bp cDNA and gDNA). These differences correspond to the prediction of introns in all genes gene (http://genome.jgi-psf.org/Mycgr3/Mycgr3.home.html; *rab*7 and *rab11* contains three introns each, *atg8* contains two introns and *cpy1* contains one intron).

We also aimed to amplify the full open-reading frame of the chitin synthase gene *mcs1*, previously used to check the quality of our RNA preparations (see above). The entire gene is predicted to contain two introns (see above, one intron locates in the 3′ end of the gene), which results in a predicted cDNA length of 5568 bps. However, using specific primers (primers CC-125 and CC-126; primers CC-127 and CC-128; Table 1), we were unable to obtain a complete PCR product. Interestingly, we obtained PCR products when amplifying the 3-prime end (1544 bp, see Table 1, primers CC-113 and CC-161; Table 1), but also the 5-prime end of the *mcs1* open-reading frame in all libraries (707 bp in gDNA; 585 bp in cDNA, primers CC-125 and CC-117; Table 1, see Fig. 2A). We started cDNA synthesis from the 3-prime end, using an oligo(dT) primer CDS III (see Section 2). Thus, the presence of the 5′ end of the *mcs1* indicates that very large cDNAs are also present in our libraries. One possibility is that our PCR failed to amplify the entire 5568 bp of the *mcs1* open-reading frame.

### 3.4 Interaction of ZtAtg4 with ZtAtg8 in an Y2H assay

Finally, we set out to test our libraries for identifying interacting proteins. In the rice blast fungus *M. oryzae*, the cysteine protease Atg4 interacts with the autophagosome-related protein Atg8 in Y2H assays (Liu et al., 2010). We considered it likely that such an interaction occurs in *Z. tritici*, too. To test this, we identified homologues of Atg4 (ZtAtg4; XP_003848416.1; 43.4% identity with *M. oryzae* Atg4) and Atg8 (ZtAtg8; XP_003855091.1; 89.4% identity with *M. oryzae* Atg8) in the *Z. tritici* published genome sequence of IPO323 at the Joint Genome Institute (http://genome.jgi.doe.gov/Mycgr3/Mycgr3.home.html). ZtAtg8 was wrongly annotated and sequence comparison with the protein from *M. oryzae* revealed that the N-terminal 432 amino acids were not part of the open reading frame. The corrected ZtAtg8 shares an autophagy protein Atg8 ubiquitin like domain with Atg8 from *M. oryzae* (*P* = 3.1e−51), and ZtAtg4 shares a peptidase family C54 domain with Atg4 from *M. oryzae* (*P* = 4.8e−98).

**Fig. 3:**
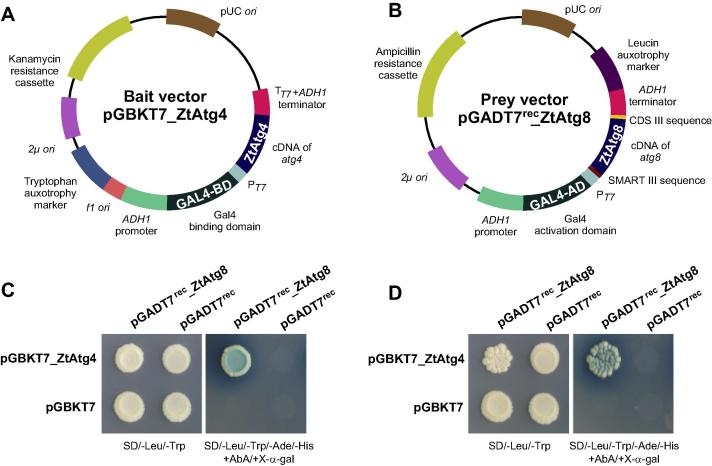
Control experiment to test the *Z. tritici* yeast two hybrid libraries. To test the quality of the IPO323_Yeasts two hybrid library, homologues of *atg4* and *atg8*, both known to interact in two hybrid experiments in other fungi (Liu et al., 2010), were cloned into the bait vector pGBKT7 (A) and the prey vector pGADT7^rec^ respectively (B; reference for both vectors (Clontech, Saint-Germain-en-Laye, France). Transformants grew in the absence of the amino acids leucine and tryptophan (-Leu, -Trp), showing that both plasmids, conferring Leu and Trp auxotrophy, were successfully transformed into the yeast cells. Under additional selection pressure (in the absence of adenine and histidine; -Ade, -His), cells can only survive if the Gal4 binding domain (GAL4-BD) interacts with the Gal4 activation domain (GAL4-AD). This occurs due to interaction of the *Z. tritici* homologues Atg4 and Atg8. In addition, such interaction triggers the expression of yeast galactosidase (MEL1), which metabolizes the chromogenic substrate X-alpha-gal (X-α-gal) and turns the colony blue. Such interaction also triggers the expression of dominant mutant version of the *AUR1* gene that encodes the enzyme inositol phosphoryl ceramide synthase, which confers resistance against Aureobasidin A. (C) Finally, we probed the yeast two-hybrid library (IPO323_Yeasts) with the bait vector pGBKT7_ZtAtg4. We found 4 potential interaction partners of ZtAtg4 that form blue colonies grow under selection pressure (-Leu, -Trp, -Ade, -His) and in the presence of X-α-gal and Aureobasidin A. Sequencing proved that all candidates were Atg8 (D, only one candidate is shown), demonstrating that the library can reveal reliable protein–protein interactions.

We cloned the full-length cDNA of ZtAtg4 and ZtAtg8 into vectors pGBKT7 and pGADT7^rec^, resulting in pGBKT7_ZtAtg4 (bait vector) and pGADT7^rec^_ZtAtg8 (prey vector), respectively (Fig. 3A and B). The plasmid pGBKT7_ZtAtg4 contains a *GAL4* DNA binding domain (GAL4-BD) fused to *atg4* as well as a yeast auxotrophic selectable marker *TRP1*, which is required for tryptophan biosynthesis (Fig. 3A). The “prey vector” pGADT7^rec^ZtAtg8 contains the *GAL4* activation domain (GAL4-AD) fused to *atg8* as well as the yeast auxotrophic selectable marker *LEU2*, which is required for leucine biosynthesis (Fig. 3B). Both vectors were co-transformed into yeast strain Y2HGold (Clontech, Saint-Germain-en-Laye, France). In addition, the following combinations were also transformed into yeast cells: (1) bait vector containing Atg4 with empty prey vector; (2) empty bait vector and prey vector containing Atg8; (3) both empty vectors. These served as controls to detect auto activation of bait and prey proteins. All yeast strains showed growth on synthetically defined (SD) media lacking tryptophan and leucine (Fig. 3C, left panel; SD/-Trp/-Leu), showing that both plasmids were transformed into the yeast cells. However, when cells were plated onto media supplemented with the anti-fungal drug Aureobasidin A and the yeast galactosidase substrate X-alpha-Gal, but lacking tryptophan, leucine, adenine, histidine, growth of blue colonies was only observed when the “bait” and the “prey” vector were co-transformed (Fig. 3C, right panel; SD/-Ade/-His/-Trp/-Leu/+X-α-Gal/+AbA). This result strongly suggests that ZtAtg4 and ZtAtg8 physically interact.

### 3.5 Screening Y2H libraries with ZtAtg4

Having established a direct interaction of ZtAtg4 and ZtAtg8, we set out to test the Y2H library, IPO323-Yeasts. We made use of the plasmid pGBKT7_ZtAtg4 and mated yeast cells Y2HGold containing this bait vector with yeast cells Y187 containing the cDNA library and plated on to SD/-Trp/-Leu/X-α-Gal/AbA plates. The mating efficiency was determined as 2.45%. After 3–5 days incubation at 30 °C, 22 blue colonies were detected, suggesting that the cells contain interacting bait and prey plasmids. This interaction was confirmed by plating these cells on more stringent plates (Fig. 3D, right panel; SD/-Ade/-His/-Trp/-Leu/+X-α-Gal/+AbA; one positive transformant was shown). In order to find out the corresponding cDNAs present on the prey vectors, we performed the PCR on four yeast transformants using primers WM12 and WM13 (Table 1) and sequenced the PCR products. This analysis revealed that all four transformants carried ZtAtg8.

## 4 Conclusion

In this study, we describe three Y2H libraries, derived from RNA isolated from two different morphological growth stages and two different wild-type isolates of *Z. tritici.* We show that the libraries are free of gDNA contamination. Random PCR shows that they contain cDNA clones of up to 2000 bp but the 5′ end of a much larger gene could be amplified, indicating that even large cDNA clones are represented in the libraries. The high-transformation efficiency suggests more than one million independent yeast colonies for each library and screening one of the libraries with ZtAtg4 as bait detected the interacting protein ZtAtg8. Thus, we conclude that all three libraries are of sufficient quality to investigate protein–protein interaction in the wheat pathogen *Z. tritici*. It should be noted that the libraries were generated as an academic community resource. They are therefore available for academic research purposes upon request.

## Figures and Tables

**Table 1:** Primers used in this study.

Primer name	Direction	Sequence (5′ to 3′)^a^
SK-Sep-63	Sense	*ATCACTCTCGGCATGGACGAGCTGTACAAG*ATGTCATCCAGAAAGAAGATCCTTT
SK-Sep-64	Antisense	*CCACAAGATCCTGTCCTCGTCCGTCGTCGC*CTAGCACGAGCAGCCTTGCTC
SK-Sep-65	Sense	*ATCACTCTCGGCATGGACGAGCTGTACAAG*ATGGCGAACGACGAATACGATGT
SK-Sep-66	Antisense	*CCACAAGATCCTGTCCTCGTCCGTCGTCGC*TCAACAGCACTGTCCGCTCTTC
SK-Sep-67	Sense	*ATCACTCTCGGCATGGACGAGCTGTACAAG*ATGCGCTCCAAGTTCAAGGACG
SK-Sep-68	Antisense	*CCACAAGATCCTGTCCTCGTCCGTCGTCGC*CTATACGGCCTCGCCGAAGGT
SK-Sep-73	Sense	*CATCACTCACATCCGCATACCACCATCGCC*ATGAAGGTCGCAGCGTCGGCC
SK-Sep-74	Antisense	*GGTGAACAGCTCCTCGCCCTTGCTCACCAT*AAACCACTCACCGCCCAACCAG
SK-Sep-75	Sense	*CATCACTCACATCCGCATACCACCATCGCC*ATGTTGGATTTCATGGACTATGTCC
SK-Sep-76	Antisense	*GGTGAACAGCTCCTCGCCCTTGCTCACCAT*GCTTGAGTAGCTGATCTCAAGAC
SK-Sep-258	Sense	*CATGGAGGCCGAATTC*AACGACTTTGCTCGCTTCAAGAAG
SK-Sep-259	Antisense	*GCAGGTCGACGGATCC*CCAACTCTCATCGTCCTCGTCC
CC-113	Sense	TTCAAGTTTCTGGCAGCACTG
CC-117	Antisense	GTACAGAACGTGAAAGTTGCG
CC-125	Sense	CATATGTCGTCCGCTCCGACTACGC
CC-126	Antisense	*GACACATTGCCGAGACCTGAAC*
CC-127	Sense	CGTGCCAGCTTCTGCGCTCGAG
CC-128	Antisense	CTCAAAGCTGACCACTGAGAATCG
CC-161	Antisense	*GCAGGTCGACGGATCC*TTGAGCGTCCCACAGCTCAGCTTG
WM3	Sense	*GGAGGCCAGTGAATTC*ATGCGCTCCAAGTTCAAGGACGAG
WM4	Antisense	*CGAGCTCGATGGATCC*CTATACGGCCTCGCCGAAGGTGT
WM12	Sense	CTATTCGATGATGAAGATACCCCACCAAACCC
WM13	Antisense	GTGAACTTGCGGGGTTTTTCAGTATCTACGATT
CDS III	Antisense	ATTCTAGAGGCCGAGGCGGCCGACATG-d(T)30VN
SMART III	Sense	AAGCAGTGGTATCAACGCAGAGTGGCCATTATGGCCGGG

a *Italics* (SK-Sep-258, SK-Sep-259, WM3 and WM4 only) indicate part of the primer that is complementary with another DNA fragment, to be ligated by In-Fusion cloning reaction. The *italics* in other primers are not relevant to this study.
